# Development of Small-Diameter Elastin-Silk Fibroin Vascular Grafts

**DOI:** 10.3389/fbioe.2020.622220

**Published:** 2021-01-14

**Authors:** Takashi Tanaka, Yasuyuki Abe, Chieh-Jen Cheng, Ryo Tanaka, Akira Naito, Tetsuo Asakura

**Affiliations:** ^1^Department of Veterinary Surgery, Tokyo University of Agriculture & Technology, Fuchu, Japan; ^2^Department of Biotechnology, Tokyo University of Agriculture & Technology, Koganei, Japan; ^3^Department of Veterinary Medicine, College of Bioresource Sciences, Nihon University, Fujisawa, Japan

**Keywords:** silk fibroin, elastin, vascular graft with small diameter, ^13^C solid-state NMR, biomaterials

## Abstract

Globally, increasing mortality from cardiovascular disease has become a problem in recent years. Vascular replacement has been used as a treatment for these diseases, but with blood vessels <6 mm in diameter, existing vascular grafts made of synthetic polymers can be occluded by thrombus formation or intimal hyperplasia. Therefore, the development of new artificial vascular grafts is desirable. In this study, we developed an elastin (EL)–silk fibroin (SF) double-raschel knitted vascular graft 1.5 mm in diameter. Water-soluble EL was prepared from insoluble EL by hydrolysis with oxalic acid. Compared to SF, EL was less likely to adhere to platelets, while vascular endothelial cells were three times more likely to adhere. SF artificial blood vessels densely packed with porous EL were fabricated, and these prevented the leakage of blood from the graft during implantation, while the migration of cells after implantation was promoted. Several kinds of ^13^C solid-state NMR spectra were observed with the EL–SF grafts in dry and hydrated states. It was noted that the EL molecules in the graft had very high mobility in the hydrated state. The EL–SF grafts were implanted into the abdominal aorta of rats to evaluate their patency and remodeling ability. No adverse reactions, such as bleeding at the time of implantation or disconnection of the sutured ends, were observed in the implanted grafts, and all were patent at the time of extraction. In addition, vascular endothelial cells were present on the graft's luminal surface 2 weeks after implantation. Therefore, we conclude that EL–SF artificial vascular grafts may be useful where small-diameter grafts are required.

## Introduction

Atherosclerosis and heart disease caused by dietary and lifestyle changes account for a significant proportion of morbidity and mortality worldwide. Vascular replacement surgery is a common procedure for such vascular diseases, and bypass surgery for revascularization is in great demand for such patients (Kibbe et al., 2010). Although vascular grafts with more than a 6 mm diameter, made of synthetic polymers, have a high patency rate (Budd et al., 1991; Takagi et al., 2010), these materials are not suitable for small-diameter (<6 mm) vascular replacement because of poor patency and occlusion. Therefore, other blood vessels, such as the internal thoracic artery, radial artery, and saphenous vein, are used for small-diameter vascular procedures (Yokota et al., 2008). Autologous vascular grafts are limited in length and diameter. Furthermore, patients who need vascular surgery often have diabetes and arteriosclerosis, and the candidate blood vessels for grafts may be impaired and cannot be harvested in many cases. Urgent operations may be impractical due to the time required to obtain the vascular grafts. Therefore, there is a need to develop a small-diameter artificial vascular graft that is easy to handle.

Silk fibroin (SF) is a natural protein produced by the silkworm and is well-known as an excellent textile material (Asakura et al., 2018). The SF fibers possess excellent mechanical properties owing to a combination of high tensile strength and breaking strain (Fu et al., 2009; Koh et al., 2015; Asakura et al., 2018). Over the past decades, significant experimental and theoretical efforts have been made to understand the relationship between the structure and the properties of the SF fibers, with some success (Fu et al., 2009; Koh et al., 2015; Pereira et al., 2015; Asakura et al., 2020a,b). This protein also has high biocompatibility, controllable biodegradability, and low toxicity. Therefore, it has also been used as a suture material for more than 2,000 years (Altman et al., 2003; Vepari and Kaplan, 2007; Thurber et al., 2015; Tamara et al., 2018; Holland et al., 2019). SF has been recently tried as a suitable candidate for vascular grafts <6 mm in diameter, as reviewed by Thurber et al. (2015), Wang et al. (2017), and by us (Asakura et al., 2019). SF vascular grafts are stiff and lacking in elasticity and need to be modified for better performance when transplanted in animals.

Elastin (EL) is an important component of the extracellular matrix of vessels. It provides elasticity in arteries, lungs, and skin (Mitchell and Niklason, 2003; Wise et al., 2009; Muiznieks et al., 2010; Baldock et al., 2011; Wang et al., 2019). In addition, EL facilitates biological functionality, i.e., minimizing platelet adhesion and reducing thrombus formation, promoting endothelial migration for angiogenesis, and regulating smooth muscle cell phenotype and proliferation (Waterhouse et al., 2011; Wang et al., 2019). Thus, EL is a very strong candidate for compensating for the shortcomings of SF blood vessel grafts. However, because of its highly crosslinked nature, EL is insoluble and difficult to use as a biomaterial (Nivison-Smith et al., 2010). Partridge et al. (1964) and Miyamoto et al. (2009) prepared water-soluble EL from insoluble EL by hydrolysis with oxalic acid. The soluble EL retains the elastic and biological properties of native EL (Miyamoto et al., 2009).

In this study, the water-soluble EL, as prepared by Miyamoto et al., was shown to improve cell adhesion and demonstrated anti-thrombogenic properties compared with SF in *in vitro* experiments. Then, a 1.5 mm diameter EL–SF artificial vascular graft was prepared by fabrication of a double-raschel knitted SF vascular graft (Yagi et al., 2011; Aytemiz et al., 2013; Fukayama et al., 2015a,b; Tanaka et al., 2018, 2020) densely packed with porous EL. The coacervation of EL, characterized by an inverse temperature transition (Wang et al., 2019), is an important prerequisite for crosslinking in preparing the EL–SF graft. The improvements in adhesion and anti-thrombogenic properties of EL were compared with SF. A comparison of the physical properties of SF-coated and EL–SF grafts was also made. Additionally, EL–SF grafts in the dry and hydrated states were studied at the molecular level using a combination of ^13^C refocused insensitive nuclei enhanced by polarization transfer (^13^C r-INEPT), ^13^C cross-polarization/magic angle spinning (^13^C CP/MAS) NMR, and ^13^C dipolar decoupled-magic angle spinning (^13^C DD/MAS) NMR (Nishimura et al., 2018; Tanaka et al., 2018). The ^13^C r- INEPT where the pulse sequence was developed for solution, NMR selectively observes the mobile components of the EL–SF graft in the hydrated state with fast isotropic motion (>10^5^ Hz) (Saitô et al., 2002). In contrast, ^13^C CP/MAS NMR selectively observes the immobile components of the samples or those with very slow motion (<10^4^ Hz) in the dry and hydrated states (Saitô et al., 2002). If the penetration of water molecules causes a local increase in the chain mobility of the graft, a loss in the CP signals occurs, and such a mobile domain cannot be observed in the ^13^C CP/MAS NMR spectra (Yang et al., 2000; Kishore et al., 2002; Holland et al., 2008). In addition, ^13^C DD/MAS NMR can be used to obtain structural information on both the mobile and immobile domains of the silk proteins in the hydrated state. Finally, the EL–SF grafts were implanted into the abdominal aorta of rats, and removed at 2, 4, and 12 weeks after implantation to evaluate their patency and remodeling ability. Based on these results, we showed that the EL–SF grafts prepared here are suitable for use as small-diameter (<6 mm) vascular grafts.

## Materials and Methods

### EL and SF

For this study, we used water-soluble EL (EL-A; molecular weight 25,200, elastic modulus >50 kPa and aggregation temperature 20–22.6°C) (extracellular matrix laboratories, Mie, Japan). The soluble EL-A sample was prepared by fractionation based on the elastic modulus and aggregation temperature of soluble EL, prepared from the insoluble EL of porcine aortas, by treatment with 0.25 M oxalic acid (Miyamoto et al., 2009). Hereafter, we refer to the water-soluble EL-A as EL in this paper.

The aqueous solution of SF was prepared as described in our previous papers (Asakura et al., 1984; Yagi et al., 2011; Saotome et al., 2015; Tanaka et al., 2018, 2020). Briefly, SF fibers obtained from cocoons were degummed in a mixture of sodium carbonate (8%, w/v) and Marseille soap (12%, w/v) at 95°C for 120 min to completely remove the silk sericin (SS). They were dissolved by adding CaCl_2_-H_2_O–EtOH solution (molar ratio: 1:8:2) to the SF at a concentration of 10% (w/v) and then boiling at 70°C for 1 h. The solution was then filtered to remove residual solid components and dialyzed with a cellulose dialysis membrane against distilled water at 4°C for 3 days to produce the aqueous solution of SF.

### Cell Adhesion Experiment

Human umbilical vein endothelial cells (HUVECs, Lonza, Inc., Switzerland) were used for the cell adhesion experiment with SF and EL- coated 24-well plates (Saotome et al., 2015). To prepare these, after EL was spread on the plates, 10% glutaraldehyde was added to crosslink the EL (Dardik et al., 2002; Osborne et al., 2008). After removing the glutaraldehyde, the surface was washed with 70% ethanol and then three times with phosphate-buffered saline (PBS). The HUVECs, cultured in endothelial basal medium-2 (EBM-2, Lonza, Inc. Switzerland), were incubated at 37°C for 1 day. The cultured HUVECs were washed with PBS, detached with 0.25% trypsin, washed, and resuspended in the medium. The HUVEC suspension was added to SF and EL-coated 24-well plates at a concentration of 2 × 10^5^ cells/well. The cells were cultured at 37°C and 5% CO_2_ for 5 h. After removing the supernatant, the living cells were stained with Calcein AM (Takara Bio Inc., Japan). The number of cells was counted using a fluorescence microscope BIOREVO BZ-9000 with BZ-H1C software (Keyence Co., Ltd., Japan). Three points were selected on each plate, and the number of cells was determined and averaged. The cultivations were performed three times with HUVEC.

### Platelet Adhesion Experiment

Dog blood in a collection tube containing citric acid was centrifuged for 15 min at 3,000 rpm. The supernatant was collected to obtain the canine platelet-rich plasma (PRP). The PRP on EL and SF- coated 24-well plates was incubated at 37°C for 3 h. After light cleaning with PBS, the EL was fixed with glutaraldehyde for 1 h at room temperature. After washing with PBS and drying, the number of platelets at three points was counted on the images with a scanning electron microscope (SEM) (VE-7800; Keyence Co., Japan) and averaged. The experiments were performed three times with PRP.

### Preparation of EL–SF Grafts

The 1.5 mm diameter double-raschel knitted silk tubes with high elongation and contraction properties were prepared with silk fibers containing reduced SS. We used a computer-controlled double-raschel knitting machine (Fukui Wrap Knitting Co., Ltd.) (Tanaka et al., 2018, 2020). Textured wefts were passed through the loops of Denbigh-stitched warps to form a structure, with the wefts as the female member of the hook-and-loop fastener. The SS in the knit tube was then removed completely by degumming as described previously (Yagi et al., 2011; Aytemiz et al., 2013).

Next, a 1.5 mm diameter PTFE rod was inserted into the SF knitted tube and immersed in a test tube filled with EL aqueous solution at 10°C for 1 h. After incubation at 50°C for 30 min to coacervate the EL, the SF knitted tube containing EL was transferred to a test tube containing the 5 w/v% glutaraldehyde aqueous solution. It was immersed for 5 min then, using 70% ethanol, it was washed and immersed overnight for sterilization. The graft was removed from the PTFE rod to be used for the next experiments. The preparation of the SF-coated SF graft used for comparison has been described (Yagi et al., 2011; Aytemiz et al., 2013; Fukayama et al., 2015a,b; Tanaka et al., 2018, 2020). The inner and outer surfaces of the SF graft and EL–SF graft were examined with the SEM. Hematoxylin and eosin (HE) staining was performed to examine the inner part of the EL–SF graft structure.

### Physical Properties of EL–SF Grafts

The circumferential tensile strengths of the SF graft, SF-coated SF graft, and EL–SF graft were determined using an EZ-graph manufactured by SIMAZU, Kyoto, Japan (Saotome et al., 2015). The road cell was 100 N, and the rate of stretching was 2 mm/min. Moreover, the circumferential compressive elastic modulus of the knitted grafts was also determined using the EZ-graph. However, we could not measure the compressive elastic modulus of the SF graft because it collapsed immediately. The road cell was 5 N, and the rate of compression was 2 mm/min. The elastic modulus (N/mm^2^), when compressed to 25% of the diameter, was calculated using the analysis software TRAPEZIUM. Ten dry, short, ring-shaped specimens with an axial length of 10 mm were prepared for the determinations, conducting four parallel tests, and the values were averaged.

### Characterization of Dry and Hydrated EL–SF Grafts With ^13^C Solid-State NMR

The ^13^C CP/MAS,^13^C DD/MAS, and ^13^C r- INEPT NMR spectra of the EL–SF grafts in the hydrated state and the ^13^C CP/MAS NMR spectrum of the grafts in the dry state were observed with a Brucker Avance 400 NMR spectrometer. After hydration overnight, the EL–SF was carefully inserted into a zirconia rotor and sealed with a PTFE insert to prevent dehydration during the NMR observations (Asakura et al., 2015a). Typical experimental parameters for the ^13^C CP/MAS NMR experiments were as follows. A 4 mm double resonance MAS probe, a MAS frequency of 8.5 kHz, 3.5 μs ^1^H 90° pulse, 1 ms ramped CP pulse with 71.4 kHz rf field strength, two-pulse phase modulation (TPPM) ^1^H decoupling during acquisition, 2,176 data points, 8 k scans, and 4 s recycle delay at room temperature. Details of the NMR experimental conditions for the ^13^C DD/MAS NMR experiments have been described earlier (Asakura et al., 2015a). Typical experimental parameters for the refocused INEPT NMR experiments included a 3.5-μs ^1^H and a 3.6-μs ^13^C 90°pulse, an inter-pulse delay of ^1^J_CH_/4 (^1^J_CH_ = 145 Hz), a refocusing delay of ^1^J_CH_/6 or ^1^J_CH_/3. The ^13^C chemical shifts were calibrated externally through the methylene peak of adamantane observed at 28.8 ppm with respect to external tetramethylsilane (TMS) at 0 ppm (Asakura et al., 1984).

### *In vivo* Evaluation of EL–SF Grafts by Implantation in a Rat

All experimental procedures and protocols were approved by the Tokyo University of Agriculture and Technology (TUAT, Approval number: 27–100). The rats were managed and cared for in accordance with the standards established by TUAT and described in its “Guide for the Care and Use of Laboratory Animals. EL–SF grafts were implanted in the abdominal aorta of 12 rats under a stereoscope (LEICA M60; Leica Microsystems GmbH-Wetzlar, Germany) (Fukayama et al., 2015a,b; Tanaka et al., 2018, 2020). Three of the rats were sacrificed at 2 weeks after implantation, six at 4 weeks, and the remaining three, at 12 weeks. The EL-coated SF grafts were 1 cm in length and 1.5 mm in inner diameter. The rats were anesthetized with intraperitoneal pentobarbital (50 mg/kg of body weight). The abdominal aorta was carefully exposed, and the aortic branches in this segment were ligated. The aorta was removed and replaced with an EL–SF graft by an end-to-end anastomosis using 9–0 monofilament nylon sutures (BEAR, Japan). The distal and, subsequently, the proximal vascular clamps were slowly removed, and flow was restored through the grafts. Graft patency was confirmed visually. No anticoagulant or antiplatelet agents were administered postoperatively. Before euthanasia, the rats were perfused with 0.9% saline solution through the left ventricle. The grafts were carefully removed together with the surrounding tissue, and a 4 mm transverse cut at the center was made. The sutured parts of the remaining native blood vessel and artificial vascular graft were cut transversely. These samples were fixed with ethanol for histological analysis. Fixed samples were embedded in paraffin and stained with HE, Masson's trichrome (MTC). Sections for immunohistochemical staining were incubated with primary antibodies, including CD31 anti-rat antibody (BD biosciences Inc. San Jose, CA, USA). These samples were incubated with N-Histofine Simple Stain Rat MAX-PO (Nichirei Biosciences Inc. Tokyo, JAPAN). Subsequently, color development was performed using the ImmPACT DAB Peroxidase Substrate Kit (Vector Laboratories Inc. Burlingame, CA, USA).

## Results

### Cell and Platelet Adhesion Experiments With SF and EL

**Figure 1:**
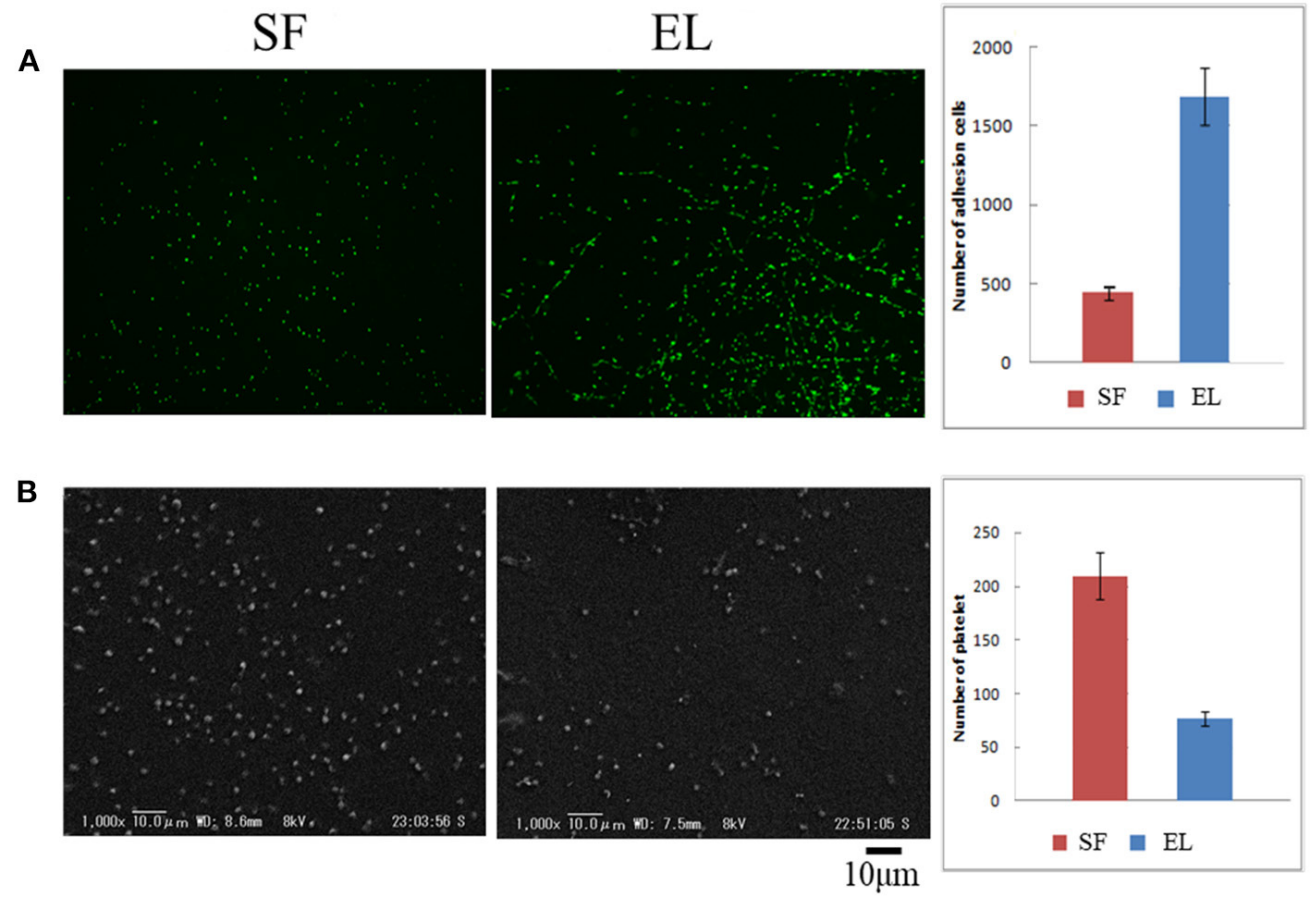
**(A)** (left) Pictures of attachment of human umbilical vein endothelial cells on silk fibroin (SF) and elastin (EL)-coated 24-well plates. (right) The number of cell attachments shown as a histogram. **(B)** (left) Scanning electron microscope images of platelet adhesion on SF and EL-coated 24-well plates (right). The number of platelet attachments shown as a histogram.

Figure 1 (**A**: left) shows the pictures of HUVEC attachment to SF and EL-coated 24-well plates. The number of HUVECs was greater for the EL-coated plate than for the SF-coated plate, 1684 ± 180, and 449 ± 54, respectively, Figure 1 (**A**: right). Thus, EL exhibited a three-fold higher attachment rate of HUBECs. Figure 1 (**B**: left) shows SEM images of platelet adhesion on SF and EL-coated 24-well plates. The attachment rate of platelets was three-fold higher for SF, compared to EL, Figure 1 (**B**: right). The platelet adhesion count for EL and SF-coated 24-well plates was 76 ± 6 and 210 ± 22, respectively. In summary, EL had a higher attachment rate for HUVECs and a lower attachment rate for platelets than SF. Therefore, EL has promising anti-thrombosis properties that may be beneficial for future applications of small-diameter grafts.

### SEM and Microscope Observations of EL–SF Grafts

**Figure 2:**
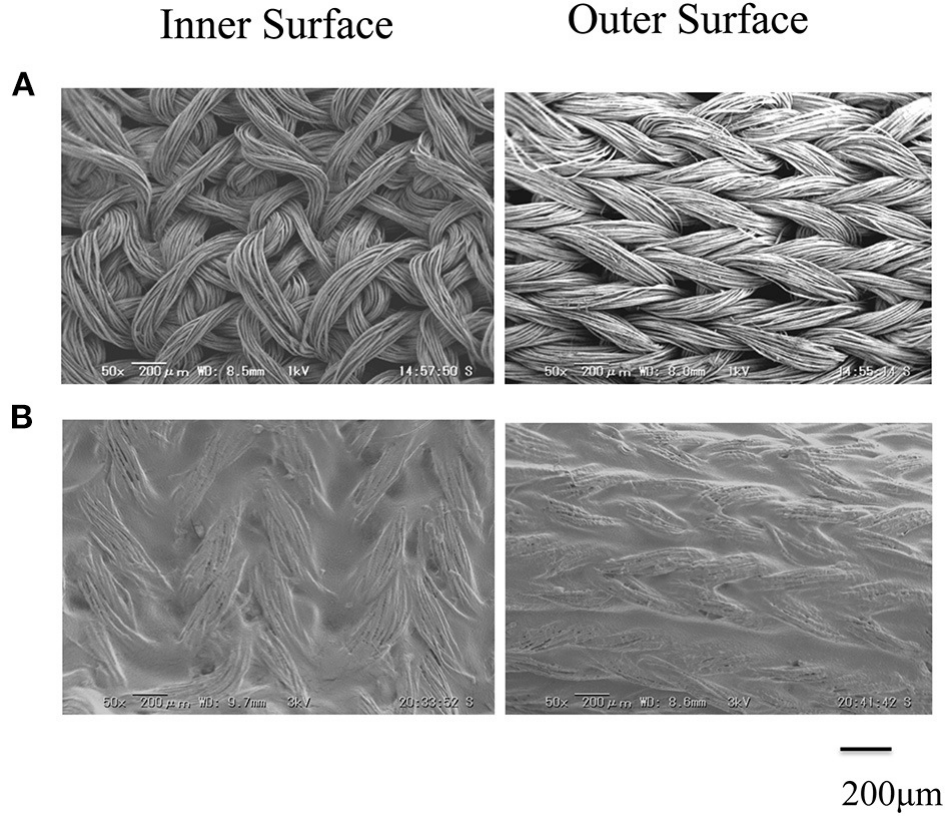
Scanning electron microscope images of **(A)** double-raschel knitted silk fibroin (SF) vascular graft and **(B)** elastin (EL)–SF knitted graft. The inner and outer surfaces of the SF graft appear to be properly coated by EL.

**Figure 3:**
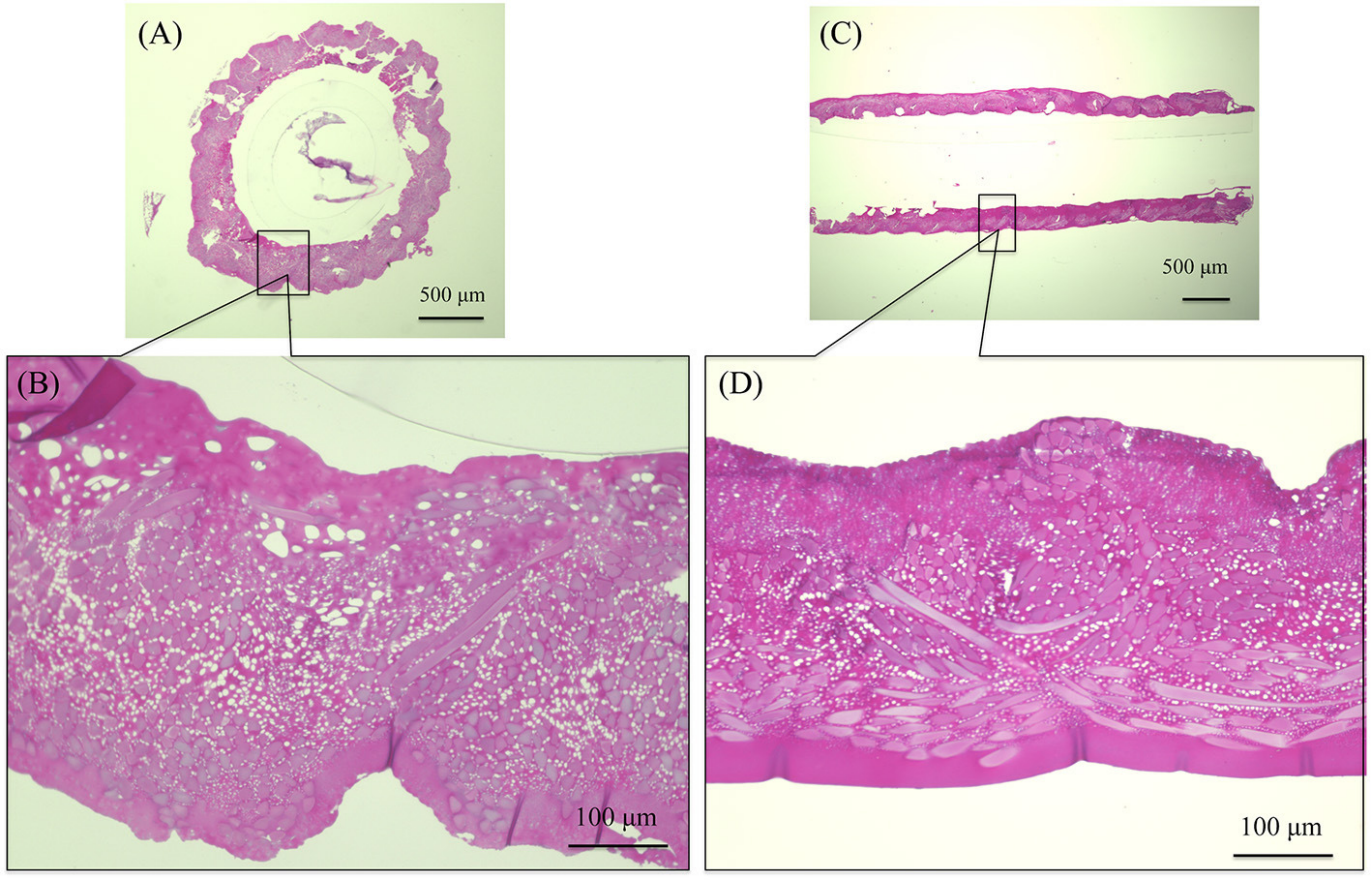
Hematoxylin and eosin staining of elastin (EL)–silk fibroin (SF) graft before implantation: **(A)** histological cross-section image and **(B)** higher magnification of the EL–SF graft, **(C)** histological longitudinal-section image and **(D)** higher magnification of the EL–SF graft. The gaps among the SF fibers in the double-raschel knitted graft were filled with a lot of spongy EL.

The SEM images are shown in Figure 2A double-raschel knitted SF graft before coated by EL. The graft prepared by the double-raschel knitting machine is very porous. Low-molecular-weight materials can easily diffuse out, and therefore too much blood would leak without a proper coating on the graft. The inner and outer surfaces of the SF graft appear to be properly coated by EL, as shown in Figure 2B. As shown in Figure 3, the HE staining of the EL–SF graft shows that the gaps between the SF fibers in the double-raschel knitted graft were filled with spongy EL. Thus, it is expected that the leakage of blood would be effectively prevented, and the infiltration of cellular tissue into the graft would be promoted.

### Physical Properties of SF- and EL- SF Grafts

**Figure 4:**
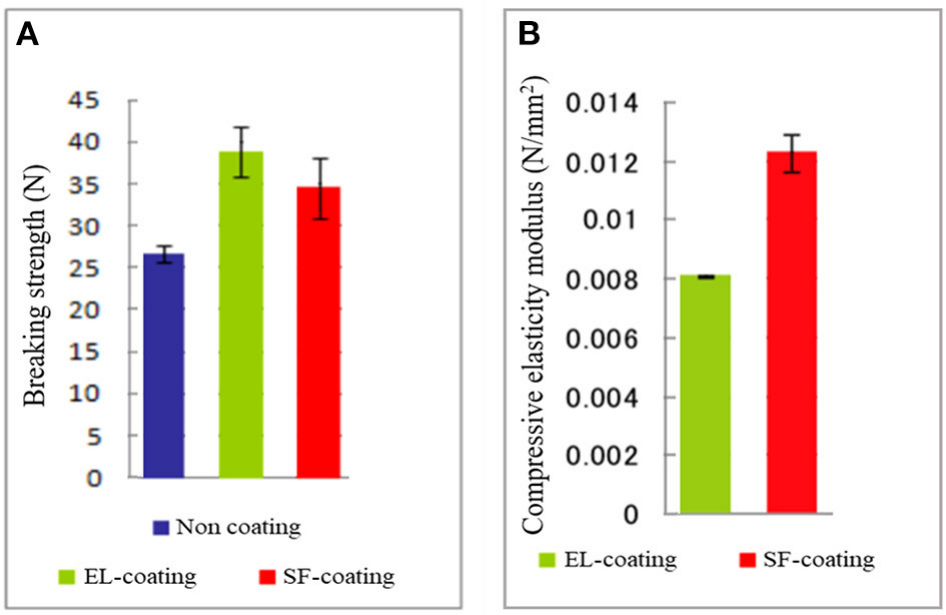
**(A)** Circumferential tensile strengths determined for non-coated, silk fibroin (SF)-coated and elastin (EL)-coated SF knitted grafts. **(B)** Circumferential compressive elastic modulus of the EL and SF-coated SF grafts. We could not measure the compressive elastic modulus of non-coated SF graft because it collapsed immediately.

A further examination of the EL–SF grafts was performed by determining the physical properties and characterization by several kinds of ^13^C solid-state NMR at the molecular level. Figure 4A shows the histogram of the circumferential tensile strengths determined for non-coated, EL–SF, and SF-coated SF grafts. The tensile strength was increased by coating, and the strength was greater for the EL–SF graft (38.0 N) than SF-coated SF graft (34.5 N). On the other hand, the circumferential compressive elastic modulus of the EL–SF graft (0.008 N/mm^2^) was lower than the SF-coated SF graft (0.012 N/mm^2^), as shown in Figure 4B. Thus, more favorable physical properties were obtained for the SF knitted graft by filling with EL sponge (Asakura et al., 2019).

### ^13^C Solid-State NMR Spectra of EL–SF Graft in the Hydrated and Dry States

**Figure 5:**
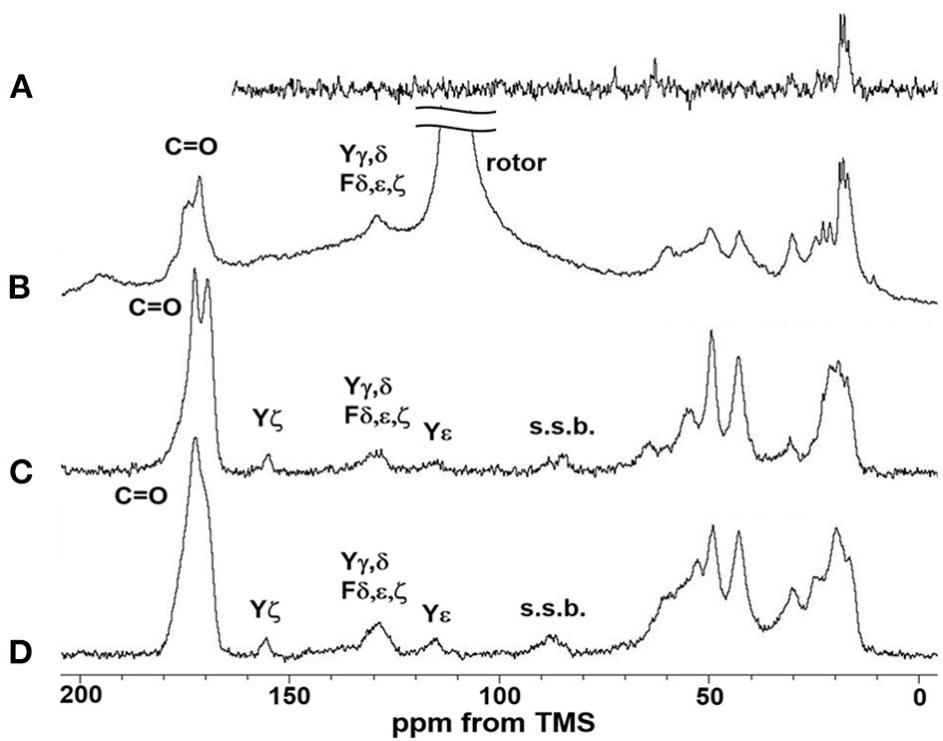
**(A)** ^13^C INEPT, **(B)** ^13^C DD/MAS and **(C)** ^13^C CP/MAS NMR spectra of EL–SF grafts in the hydrated state together with **(D)** ^13^C CP/MAS NMR spectrum of elastin–silk fibroin graft in the dry state. Only the field lower than 80 ppm was assigned in this figure and the field higher than 80 ppm was assigned in Figure 6. The largest peak in **(B)** is due to rotor and the peaks s.s.b.in **(C)** and **(D)** are due to spinning side band.

**Figure 6:**
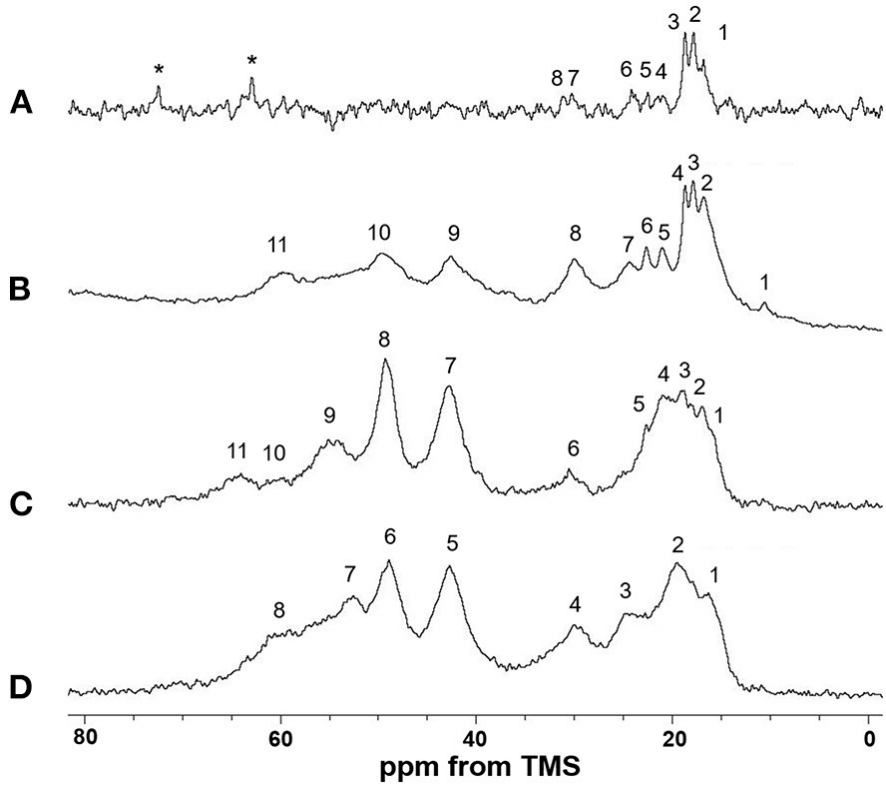
Expanded (0–80 ppm) regions of **(A)** ^13^C INEPT, **(B)** ^13^C DD/MAS and **(C)** ^13^C CP/MAS NMR spectra of elastin (EL)–silk fibroin (SF) grafts in the hydrated state with **(D)** ^13^C CP/MAS NMR spectrum of EL–SF graft in the dry state. The assignment of the peaks numbered in the spectra is summarized in Table 1. Two peaks marked by *in **(A)** are due to unidentified impurities. The Ala Cβ peak with anti-parallel β-sheet structure from SF is considered to be the main peak at around 20 ppm in the range from 15 to 25 ppm in **(C)**.

Figure 5 shows the (A) ^13^C r-INEPT, (B) ^13^C DD/MAS, and (C) ^13^C CP/MAS NMR spectra of EL–SF grafts in the hydrated state, and (D) shows the ^13^C CP/MAS spectrum of the graft in the dry state. The expanded region (0–80 ppm) of these spectra are also shown in Figures 6A–D. The ^13^C chemical shifts and assignments are listed in Table 1.

**Table 1:** ^13^C chemical shifts of ^13^C solid-state NMR spectra of EL-SF graft.

**^**13**^C solid-state NMR (EL-SF Graft)**	**Number (Figure 6)**	**Chemical shift (ppm)**	**Assignment**	
			**EL**	**SF**
(a)INEPT(hydrated)	1	16.8	Aβ(r.c.)	
	2	17.9	Vγ_2_	
	3	18.7	Vγ_1_	
	4	21.2	Lδ_2_	
	5	22.6	Lδ_1_	
	6	24.1	Pγ + Lγ	
	7	30.0	Pβ	
	8	31.1	Vβ	
(b) DDMAS(hydrated)	1	10.7	Iδ	
	2	16.8	Aβ(r.c.)	
	3	17.8	Vγ_2_	
	4	18.7	Vγ_1_	
	5	21.1	Lδ_2_	
	6	22.8	Lδ_1_	
	7	24.4	Pγ+Lγ	
	8	30.0	Pβ+Vβ	
	9	42.8	Gα	
	10	49.4	Aα(r.c.)+Pδ	
	11	59.4	Vα + Pα	
		129.2	Yγ,δ + Fδ,ε.ζ	
(c) CP/MAS(hydrated)	1	16.9	Aβ(r.c.)	
	2	18.0	Vγ_2_	
	3	19.1	Vγ_1_	Aβ(β-sheet; 20 ppm)
	4	21.1	Lδ_2_	
	5	22.7	Lδ_1_	
	6	30.7	Vβ	
	7	42.6	Gα	Gα(β-sheet)
	8	49.2	Aα(r.c.+β-sheet)	Aα(β-sheet)
	9	54.4		Sα(β-sheet)
	10	60.3	Vα+Pα	
	11	64.2		Sβ(β-sheet)
		114.9	Yε	
		128.6	Yγ,δ+Fδ,ε,ζ	
		155.0	Yζ	
(d) CP/MAS(dried)	1	16.6	Aβ(r.c.+α-helix)	
	2	19.3		Aβ(β-sheet; 20 ppm)
	3	24.3	Pγ+Lγ	
	4	29.6	Pβ+Vβ	
	5	42.5		Gα(β-sheet)
	6	49.2	Aα(r.c.+β-sheet)	Aα(β-sheet)
	7	52.8	Aα(α-helix)	
	8	60.1	Pα	
		114.9	Yε	
		128.7	Yγ,δ+Fδ,ε,ζ	
		155.2	Yζ	

*r.c., random coil*.

The sharp peaks observed in the ^13^C r-INEPT spectrum, Figure 5A, were assigned to only the ^13^C nuclei with fast motion in the hydrated EL–SF graft (Tanaka et al., 2018). There are no peaks in the carbonyl region because no ^1^H nuclei are attached to the carbonyl carbons directly in the ^13^C r-INEPT spectrum. The ^13^C r-INEPT spectrum of SF fibers in the hydrated state reported previously (Tanaka et al., 2018) showed sharp random coil peaks of Ala Cα, Ala Cβ, Gly Cα, Ser Cα, and Ser Cβ carbons. However, these peaks from SF fibers were not seen in the ^13^C r-INEPT spectra of the EL–SF graft, Figures 5A, 6A. The hydration of the SF chain is suppressed because of the hydrophobic environment around it due to the presence of EL molecules and the crosslinking with EL molecules through the amino groups of the Lys side-chain by glutaraldehyde (Wise et al., 2005). Three peaks, i.e., two Val peaks, VC_γ1_ and VC_γ2_, and one Ala Cβ random coil peak could be observed together with very small Val Cβ, Pro Cβ, Pro Cγ, Leu Cδ_1_, and Leu Cδ_2_ peaks from EL (Figure 6A and Table 1) (Wuethrich, 1976; Asakura et al., 1984; Ohgo et al., 2009; Papaioannou et al., 2015). Two sharp peaks at 63 ppm and 72 ppm are unidentified peaks from impurities.

As shown in Figure 5B, a single large peak at 110.8 ppm was observed from the rotor (Teflon) in the ^13^C DD/MAS NMR spectrum of the hydrated EL–SF graft. The peak at 129.2 ppm was assigned to aromatic carbons from Tyr (Cγ and Cδ carbons) and Phe (Cδ, Cε and Cζ carbons) residues (Table 1). The carbonyl peaks were also observed in the range of 171–175 ppm. There are many other peaks in the 10–60 ppm range. These peaks are considered to be due to the carbons in the EL molecules, although the peaks from both EL and SF can be seen. If the peaks from SF were observed in Figure 5B, the Ala Cβ peak from SF should be observed at about 20 ppm (Asakura et al., 1999, 2015b; Holland et al., 2008; Nishimura et al., 2018). However, the Ala Cβ peak could not be seen because of the significantly restricted motion of the SF fibers. The Ala Cβ peak of the SF fibers could be observed in the ^13^C CP/MAS NMR spectra, Figures 5C, 6C, of the hydrated EL–SF graft as the broad peak in the range of 15–25 ppm together with the Gly Cα, Ala Cα, Ser Cα, and Ser Cβ peaks from SF fiber with AP-β structure as listed in Table 1 (Asakura et al., 1999, 2015b; Holland et al., 2008; Nishimura et al., 2018). By considering both the amino acid compositions of EL and the ^13^C chemical shifts of the peaks, the ^13^C DD/MAS NMR spectrum could be assigned as shown in Figure 6B and listed in Table 1. Thus, the EL molecules in the EL–SF graft are relatively mobile in the hydrated state. As mentioned above, the peaks from the SF fibers were clearly observed in Figures 5C, 6C, and conversely, the peaks from EL became relatively small. This is due to a significant loss in CP signals of the carbons in EL molecules whose mobilities are very high (Yang et al., 2000; Kishore et al., 2002; Holland et al., 2008; Nishimura et al., 2018). The Pro and Leu peaks, in particular, could not be observed in the ^13^C CP/MAS NMR spectrum of the hydrated graft, indicating the high mobility of these residues. The presence of the Pro residues in the primary structure is thought to be a source of higher hydration of the pro-containing protein fibers such as spider silk fiber and elastin fiber (Asakura et al., 2003; Ohgo et al., 2005, 2018; Jenkins et al., 2010; Muiznieks et al., 2015; Dabalos et al., 2019).

Most of the peaks from EL could be seen in the dry state along with the peaks from the SF fibers, as shown in Figures 5D, 6D. The Pro Cα, Pro Cβ, and Pro Cγ peaks together with Val Cα, Val Cβ, and Leu Cγ peaks from EL were newly observed (Table 1) (Wuethrich, 1976; Asakura et al., 1984; Ohgo et al., 2005, 2009, 2018; Papaioannou et al., 2015). The peak at 52.8 ppm could be assigned to the Cα peak of Ala residues with the α-helical form of EL molecules judging from the chemical shift (Asakura et al., 1999). The presence of the α-helical structure of Ala residue has been reported for elastin samples (Papaioannou et al., 2015; Ohgo et al., 2018). Thus, a slightly higher field shift of Ala Cβ peaks compared with the Ala Cβ chemical shifts of the other three NMR spectra (Figures 6A–C) in the hydrated state indicates a mixture of α-helix (15.8 ppm) and random coil (16.8 ppm) conformations. The Ala Cα random coil peak (50.3 ppm) is included in the AP-β Ala Cα peak (49.2 ppm). From these NMR data, the high mobility of the EL molecules in the EL–SF graft in the hydrated state became clear, which is expected to be fully functional of EL shown in Figure 1, i.e., minimizing platelet adhesion and promoting endothelial migration.

### *In vivo* Experiment With the EL–SF Graft

**Figure 7:**
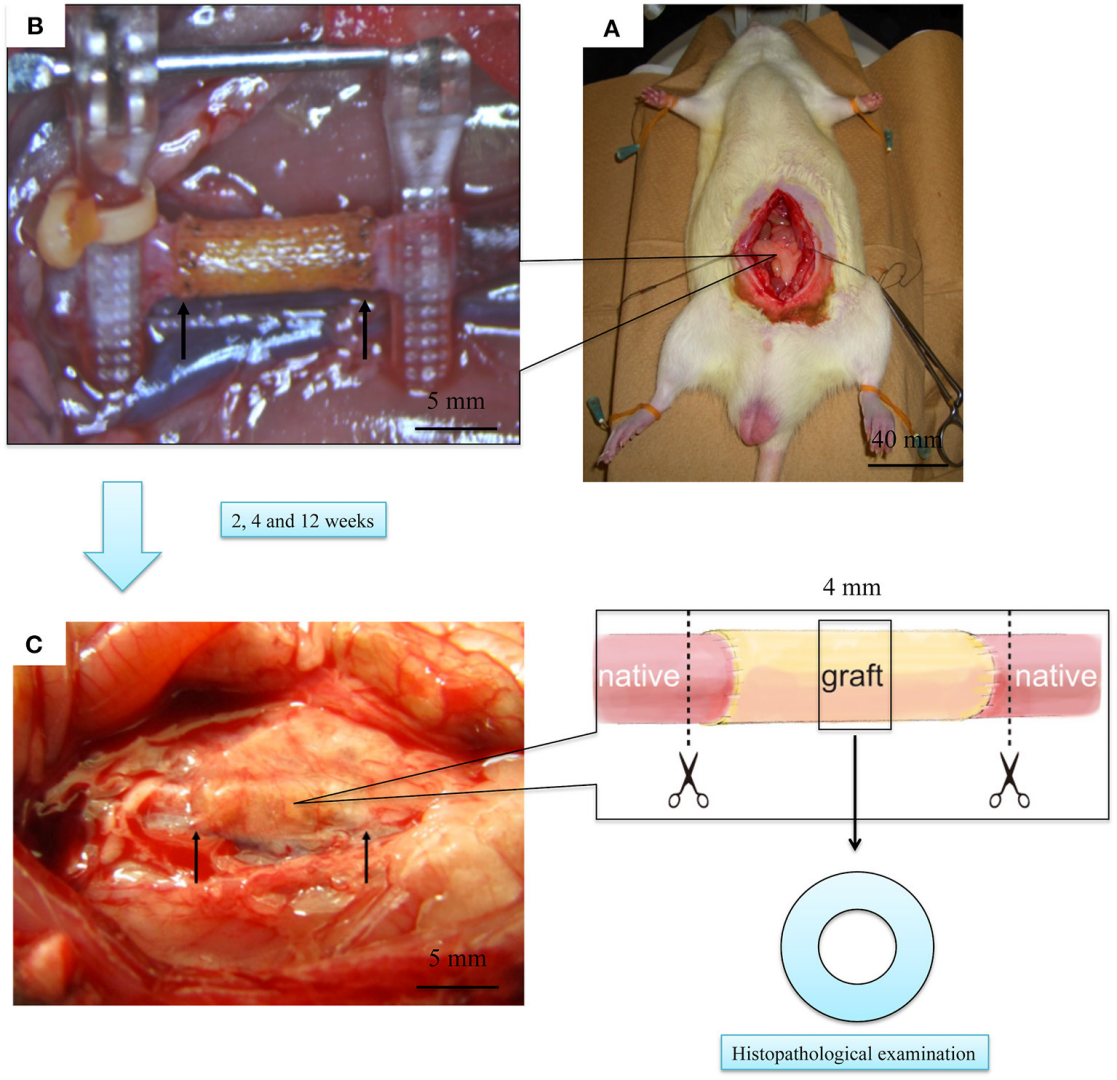
Laparotomy images of the rat implantation experiment of elastin (EL)–silk fibroin (SF) graft. **(A)** Picture of the graft immediately after transplantation. The two yellow arrows indicate the cranio-caudal anastomosis of the graft. **(B)** Picture of the rat with open abdomen and transplanted EL–SF graft. **(C)** Picture of removing artificial vascular graft at 2 weeks after implantation. The grafts, including autologous blood vessels, were removed using scissors at the dotted line.

After implanting the EL–SF grafts (Figure 7), all rats were confirmed to have good blood flow. In addition, we confirmed that the tissue was attached sufficiently when all grafts were removed. The grafts were mainly covered by fat and infiltrated blood vessels were also observed, Figure 7C. The adipose tissue could be easily detached from the grafts with a cotton swab. The formation of granuloma, which is a foreign body reaction, did not occur. We, therefore, consider that SF and EL used for transplantation were not harmful to rats.

**Figure 8:**
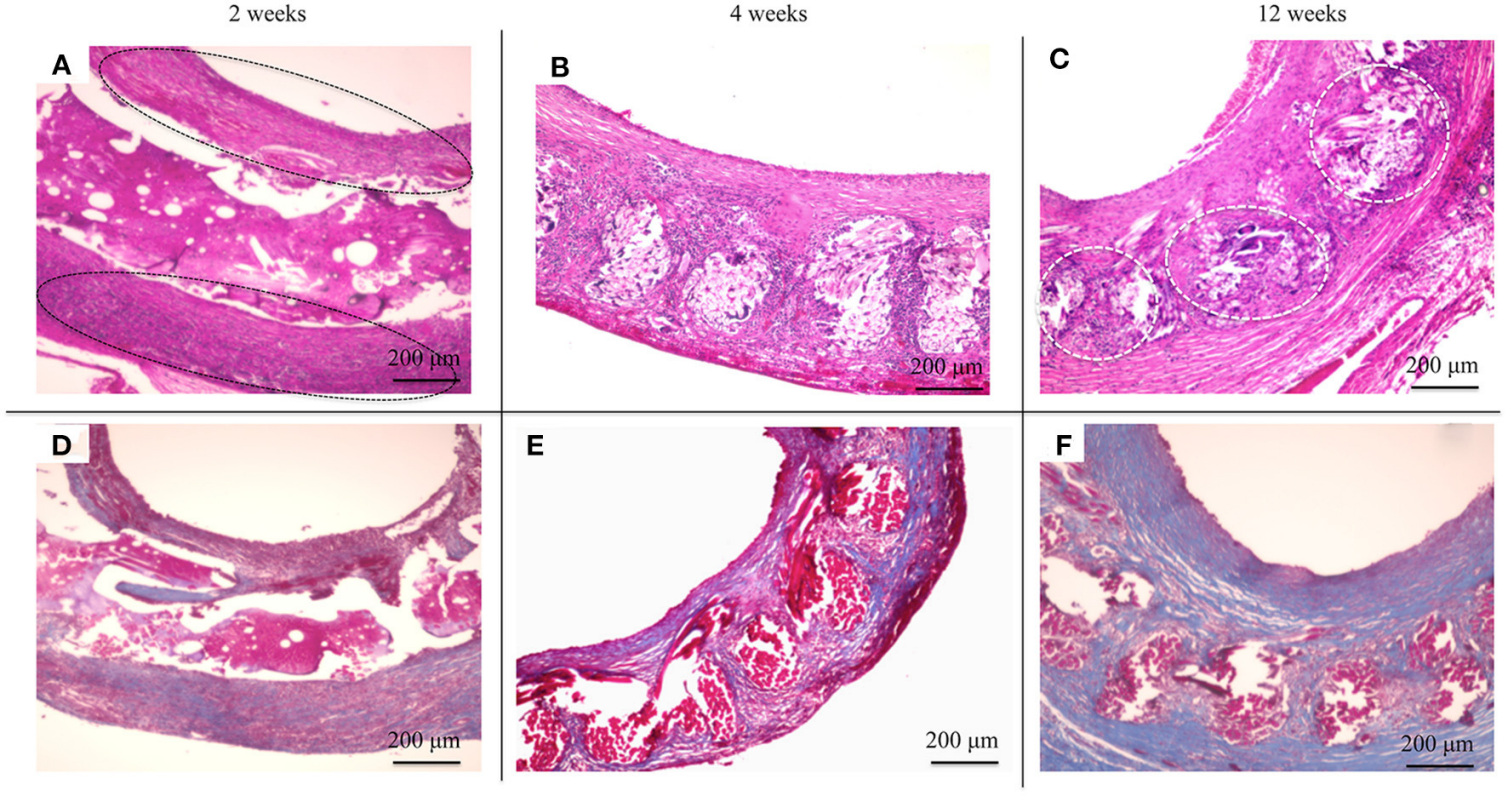
Histological cross-section images at 2 weeks after implantation: **(A)** hematoxylin and eosin staining (HE) and **(D)** Masson's trichrome (MTC) staining. Histological cross-section images at 4 weeks after implantation: **(B)** HE staining and **(E)** MTC staining. Histological cross-section images at 12 weeks after implantation: **(C)** HE staining and **(F)** MTC staining. The black circles indicate the inflammatory cells gathered in layers **(A)**, and the white circles indicate fibroblasts and macrophages had invaded the interstices of the SF fibers **(C)**.

**Figure 9:**
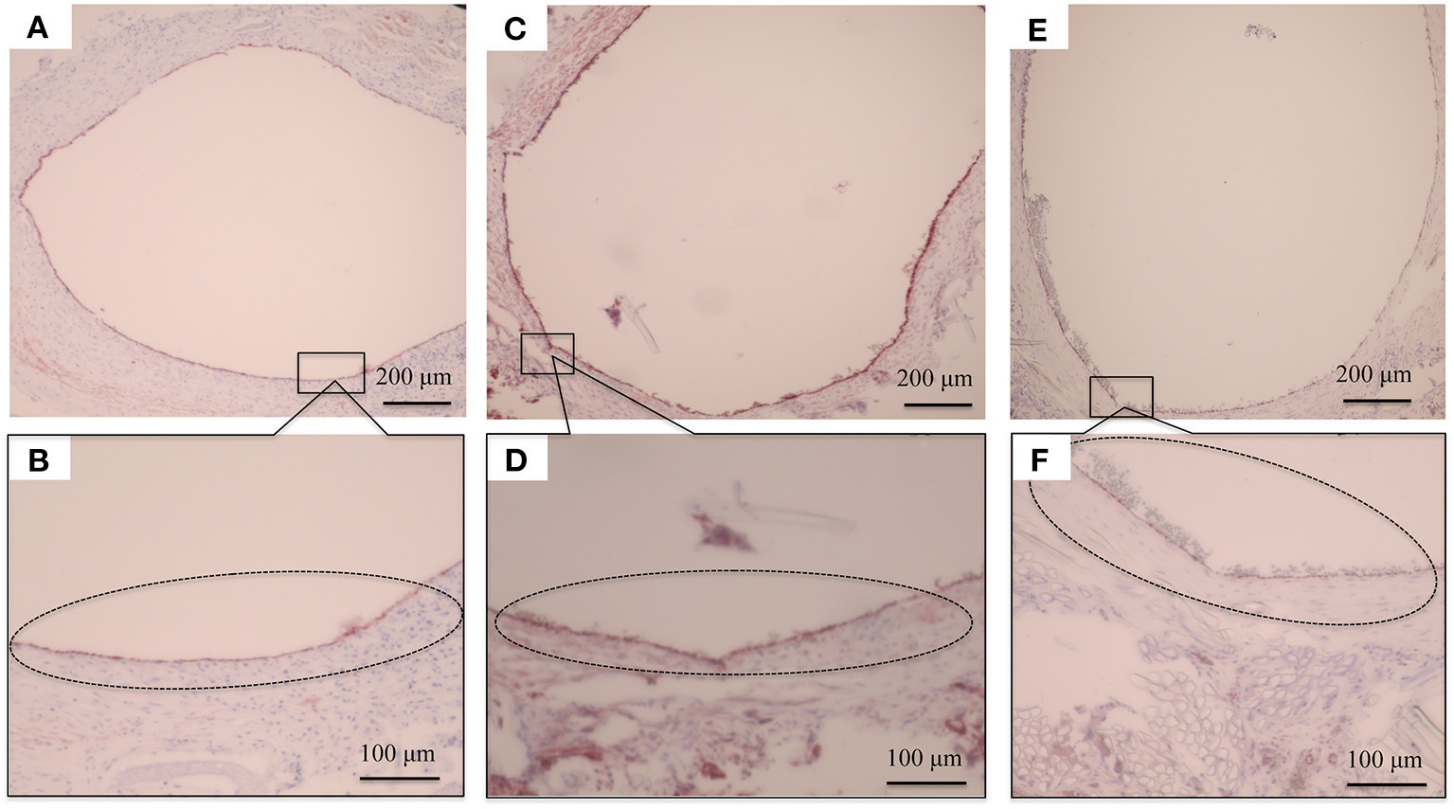
Histopathological photographs of CD31 staining at **(A)** 2 weeks after implantation and **(B)** higher magnification. **(C)** CD31 staining at 4 weeks after implantation and **(D)** higher magnification. **(E)** CD31 staining at 12 weeks after implantation **(F)** and higher magnification. The black circles indicate the vascular endothelial cells adhering to the luminal surface of the graft.

HE staining was used to investigate how the implanted graft changed after implantation. At 2 weeks, there was no cell infiltration into the grafts because the EL sponge had not decomposed (Figure 8A). Since EL molecules filled the gaps between the SF fibers in the grafts, the SF fibers and the filled EL were seen to form a single structure. Inflammatory cells such as neutrophils, lymphocytes, macrophages, and fibroblasts were mainly gathered inside and outside the graft. At 4 weeks after implantation, it was confirmed that the EL was roughly decomposed, and fibroblasts and macrophages invaded the gaps between SF fibers in the graft. The numbers of inflammatory cells accumulated inside and outside the grafts were reduced compared to 2 weeks after implantation, Figure 8B. A layered structure observed in the graft's center was confirmed to have an aggregation of vascular smooth muscle cells and collagen fibers within it. At 12 weeks after implantation, the EL could not be detected, and many fibroblasts and macrophages had invaded the interstices of the SF fibers (Figure 8C). The collagen fibers were dyed blue with MTC staining to examine their involvement in artificial vascular graft remodeling after implantation. The collagen fibers were sparsely present outside and inside the grafts at 2 weeks after implantation (Figure 8D). Since the EL molecules remained inside the graft, the collagen fibers did not initially invade, however, the numbers around the graft increased over time. The collagen fibers also penetrated into the SF fibers (Figure 8E). At 12 weeks after implantation, the number of collagen fibers inside and around the graft increased to cover the entire graft. Further, collagen fibers could be confirmed inside the graft with a concentric, layered formation (Figure 8F). The immunohistochemical staining results confirmed the endothelialization of the central part of the graft as early as 2 weeks. Although it could not be confirmed on part of the luminal surface, most of it adhered to the luminal surface without peeling off from the graft (Figures 9A,B). At 4 weeks after implantation, vascular endothelial cells had completely covered the graft's lumen, which remained unchanged at 12 weeks (Figures 9C–F).

## Discussion

We developed a small-diameter artificial vascular graft using SF (Enomoto et al., 2010; Yagi et al., 2011; Aytemiz et al., 2013; Fukayama et al., 2015a,b; Tanaka et al., 2018, 2020). SF has a long history of biocompatibility having been used as a surgical suture for many years (Altman et al., 2003; Vepari and Kaplan, 2007; Thurber et al., 2015; Tamara et al., 2018). It is known to be biodegradable *in vivo*, and its shape and properties can be controlled by various processing methods (Horan et al., 2005; Enomoto et al., 2010; Numata et al., 2010; Guo et al., 2020).

Elastin is found in the extracellular matrix of the intima of blood vessels. It accounts for 50% of the dry weight of arteries, and its properties include endothelial cell compatibility, blood compatibility, and elasticity. It is also attracting attention as an artificial vascular graft combined with other biomaterials (Wilson et al., 2011; Wang et al., 2019). However, because of its highly crosslinked nature, EL is insoluble and difficult to use as a biomaterial (Nivison-Smith et al., 2010).

In this study, we used water-soluble EL prepared from insoluble native elastin by hydrolysis with oxalic acid to improve SF vascular grafts. The beneficial characteristics of EL, i.e., higher attachment of endothelial cells and lower attachment of platelets, were still maintained compared with SF. Thus, we conducted an experiment to develop an EL–SF artificial vascular graft with high biocompatibility to promote endothelialization, antithrombogenicity, and elasticity. Since thrombus formation is the primary cause of occlusion in <6 mm diameter grafts (Begovac et al., 2003; Venkatraman et al., 2008; Tatterton et al., 2012), the presence of EL in the graft provided effective antithrombogenicity. It is also important to prevent leakage of blood during transplant surgery and for the graft to have good physical properties and operability (Salacinski et al., 2001). HE staining of the EL–SF graft showed that spongy EL filled the inner parts, as shown in Figure 3. This prevents blood leakage after implantation and improves the physical properties, including graft operability. We found the blood leakage was mild at the time of implantation. Conventional synthetic polymer materials cause intimal thickening after implantation due to a mismatch in compliance with the autologous blood vessels (Ballyk et al., 1997; Kashyap et al., 2002; Heise et al., 2006); however, the EL–SF graft is expected to solve the compliance difference due to its higher elasticity. We also experienced no loosening due to the needle and anastomosis, indicating improved handling during surgery.

The various ^13^C solid-state NMR spectra of the EL–SF graft in the dry and hydrated states enabled the characterization of the local structure and dynamics of EL and SF molecules at the molecular level. The hydration of the SF chains of the EL–SF graft is suppressed because of the crosslinking with EL molecules and their creation of a hydrophobic environment around the SF chains. On the other hand, the EL molecules in the EL–SF graft are highly mobile in the hydrated state, especially for the Pro and Leu residues. Thus, the EL properties effectively minimize platelet adhesion and promote endothelial migration in the EL–SF graft.

After implantation, patency was confirmed in all EL–SF grafts. No signs such as hematuria, diarrhea, inflammation, tail disintegration, or leg immobility were observed in the rats. The EL molecules on the SF fibers should be antithrombotic, as EL is first exposed to blood when flow resumed (Fernández-Colino et al., 2019). Since thrombus was not formed, and no occlusion occurred before the EL–SF graft was covered with the vascular endothelial cells, the usefulness of the EL was shown. 2 weeks after the transplantation, many inflammatory cells such as neutrophils and macrophages infiltrated the graft's periphery. However, the accumulation of excessive inflammatory cells and encapsulation by collagen fibers, a type of foreign body reaction, did not occur. It has been reported that inflammation in the transplanted site peaks 7–14 days after the implantation, and decreases as remodeling occurs (Thurber et al., 2015). Since the inflammatory cell numbers decreased at 4 and 12 weeks after the implantation, the implanted EL–SF graft did not cause an abnormal immune response. As shown in Figure 9, the endothelialization of the EL–SF graft occurred rapidly, within 2 weeks of implantation. In addition, the endothelial cells in the lumen became organized without detachment at 4 weeks, suggesting that the EL–SF graft may prevent the blood flow from detaching the endothelial cells. Our results confirm that two factors cause endothelialization in the short term. Firstly, the proliferation and adhesion of endothelial cells starts from the anastomosis and is promoted by EL (Noishiki et al., 1995). Secondly, the endothelial progenitor cells (Asahara et al., 1997) contained in blood migrate to the lumen of the artificial blood vessel due to the decomposition of the EL. Adhesion to the coated surface and organizing by proliferation then occurs. Most of the luminal surface at 2 weeks after implantation was endothelialized, which is earlier than it occurs in SF grafts coated with SF, as demonstrated in earlier studies (Fukayama et al., 2015a,b). Thus, it is likely that dissolved EL was involved after implantation. However, tissue infiltration is unlikely to occur due to the thickness of the coating, and it will be necessary to devise ways to facilitate it. Specifically, a method for thinning the outer coating, which does not reduce the antithrombotic and endothelialization characteristics of EL in the graft's inner coating is needed.

The use of a crosslinking agent for glutaraldehyde was not a major problem in this study because it was implanted in rats. For humans, however, it is important to use low-inflammatory elastin (Wise et al., 2011) or a method that does not use a crosslinking agent (Heidenhain et al., 2011; Vasconcelos et al., 2012). In addition, a biopolymer component that has the strength of SF and the elasticity of EL has recently been developed. The researchers performed protein blending on SF with recombinant DNA (Dinjaski and Kaplan, 2016). The fibers obtained with the above technique can be used as the basis for an artificial blood vessel with better physical properties and early remodeling to self-organization. Based on these improvements, long-term artificial vascular graft implantation evaluation in large animals can be carried out, and finally, the development of clinically applicable small-diameter artificial vascular grafts may be achieved.

## Conclusion

In this study, we developed an EL–SF 1.5 mm diameter double-raschel knitted vascular graft. The SF graft was densely packed with porous EL, which prevented blood leakage from the graft during implantation. The migration of cells after implantation was promoted. The ^13^C solid-state NMR studies indicated that the EL molecules in the graft have very high mobility in the hydrated state. This graft showed excellent biocompatibility *in vitro*, and excellent operability and remodeling ability *in vivo*.

## Data Availability Statement

The raw data supporting the conclusions of this article will be made available by the authors, without undue reservation.

## Ethics Statement

The animal study was reviewed and approved by Tokyo University of Agriculture and Technology.

## Author Contributions

TA and YA designed the study. TT, YA, and AN performed experiment. All authors contributed to the article and approved the submitted version.

## Conflict of Interest

The authors declare that the research was conducted in the absence of any commercial or financial relationships that could be construed as a potential conflict of interest.
